# Intraoperative transit-time flowmetry of the superficial temporal artery directly predicts and may mitigate risk of postoperative hyperperfusion syndrome in moyamoya bypass surgery

**DOI:** 10.3389/fneur.2026.1863863

**Published:** 2026-08-17

**Authors:** Yunyu Wen, Zhibin Wang, Yucheng Wang, Fangzhou Chen, Tinghan Long, Shichao Zhang, Peng Li, Huibin Kang, Guozhong Zhang, Mingzhou Li, Wenfeng Feng, Songtao Qi, Gang Wang

**Affiliations:** 1 Department of Neurosurgery, Nanfang Hospital, Southern Medical University, Guangzhou, Guangdong, China; 2 Institute of Brain Disease, Nanfang Hospital, Southern Medical University, Guangzhou, Guangdong, China; 3 Laboratory for Precision Neurosurgery, Nanfang Hospital, Southern Medical University, Guangzhou, Guangdong, China

**Keywords:** cerebral hyperperfusion syndrome, EC-IC bypass, intraoperative flow monitoring, moyamoya disease, superficial temporal artery, transit-time flowmetry

## Abstract

**Objective:**

To obtain perioperative cerebral blood flow measurements and assess the role of intraoperative blood flow evaluation in predicting postoperative complications.

**Methods:**

We conducted a retrospective analysis of 102 cases of extracranial-intracranial (EC-IC) bypass surgery performed for flow augmentation in patients with moyamoya disease. The study monitored blood flow in the donor artery, graft vessels, and recipient artery at various stages. We measured superficial temporal artery (STA) flow during the perioperative period using a transit time ultrasonic flowmeter and Doppler ultrasound for real-time monitoring. A modified MBC scale was developed to evaluate the vascular network of the middle cerebral artery (MCA). STA flow measurements and the modified MBC scale were analyzed for their correlation with the incidence of cerebral hyperperfusion syndrome (CHS).

**Results:**

A total of 102 hemispheres underwent revascularization through EC-IC bypass, comprising 69 direct bypass cases and 33 combined bypass cases. We observed fluctuations in donor vascular flow during the perioperative period. The STA flow increased post-anastomosis, stabilizing at an elevated level thereafter. Specifically, the STA *in situ* flow measured 7.06 ± 3.30 mL/min, while the STA-Cut flow was 59.75 ± 37.49 mL/min. The flow following STA anastomosis was 36.07 ± 22.59 mL/min. Prior to the anastomosis of the bonnet aponeurosis and skin, STA flow was recorded at 35.14 ± 22.93 mL/min. Postoperative STA flow on day one was 111.91 ± 62.06 mL/min, and on day seven it was 104.47 ± 64.93 mL/min. Notably, 12.74% of patients developed CHS. Logistic regression analysis indicated that the occurrence of CHS was significantly correlated with STA-Cut flow, suggesting that lower flow rates are associated with a higher likelihood of CHS. Additionally, surgical approach and the MBC scale were also related to the incidence of CHS.

**Conclusion:**

The transit time flow (TTF) measurements indicate that the superficial temporal artery (STA) transitions from a low-flow vessel *in situ* to a medium- to high-flow graft vessel immediately after anastomosis. The variability in flow within the graft vessel plays a significant role in the occurrence of postoperative hyperperfusion syndrome.

## Introduction

Moyamoya disease (MMD) is a chronic, progressive cerebrovascular disorder characterized by bilateral stenosis or occlusion of the intracranial carotid arteries, accompanied by the development of fragile collateral vessels at the base of the brain (1, 2). Clinically, MMD manifests through transient ischemic attacks (TIAs), ischemic strokes, and intracranial hemorrhage, with patients experiencing progressive neurological and cognitive decline (3). Unfortunately, there is no effective medical treatment for MMD, making surgical revascularization—either direct, indirect, or combined bypass—the preferred therapeutic approach (4, 5).

Direct bypass surgery, where the superficial temporal artery (STA) is frequently used as the donor vessel, is a primary treatment option for moyamoya disease. The STA has traditionally been considered a low-flow vessel, with previous studies, consistent with the conventional view. However, postoperative data at 1 and 7 days suggest a significant increase in STA flow. Whether this increase occurs immediately during surgery or is part of a gradual process remains uncertain. While Charbel’s studies provide insight, their limited sample size and lack of perioperative graft flow data underscore the need for larger-scale observations (6).

Cerebral hyperperfusion syndrome (CHS) is a recognized complication following bypass surgery for MMD, but its exact incidence and the associated risk factors remain incompletely defined (7–9). Currently, intraoperative blood flow monitoring is performed using various techniques, including perivascular probes and cerebral oxygenation monitoring methods (10, 11). One commonly used device is the flexible perivascular flow probe (Charbel probe; Transonic Systems, Inc.), which can be placed around both donor and recipient vessels to measure flow in milliliters per minute. This tool provides critical data on the function of the graft, flow direction (either forward or retrograde), and allows for the adjustment of intraoperative blood pressure and postoperative hemodynamics.

Lee et al. (12) analyzed hemodynamic parameters in 292 MMD patients using the perivascular flow probe and found a fivefold increase in blood flow through the middle cerebral artery post-anastomosis (12, 13). Excessively high blood flow in this artery was linked to postoperative complications, such as ischemic stroke, hemorrhage, and transient neurological deficits, highlighting the importance of intraoperative flow monitoring for predicting complications. In patients identified as high-risk, tighter postoperative blood pressure control may be necessary.

Other methods of assessing cerebral blood flow (CBF) or oxygenation have been reported in the literature (14). Takeuchi et al. employed a thermal diffusion flow probe to measure local CBF in 14 cortical regions of 12 young MMD patients, both before and after bypass (15). Their results showed no significant reduction in cortical perfusion during temporary occlusion. Local CBF increased immediately after bypass and remained elevated in four of the eight hemispheres studied, with significant increases observed within the first 1–2 min and 5–10 min post-anastomosis. These alternative markers, along with infrared cerebral surface monitoring, provide valuable insight into the local hemodynamics after revascularization.

Perioperative alterations in cerebral hemodynamics have been increasingly recognized as potential contributors to postoperative neurological complications. However, reliable methods for intraoperative CBF assessment remain limited. Therefore, this study aimed to obtain perioperative CBF measurements using a practical and reproducible technique, and further to evaluate whether intraoperative CBF evaluation could help predict postoperative complications. By establishing a quantitative CBF parameter, we sought to identify high-risk patients earlier and potentially guide perioperative management strategies.

## Patients and methods

### Patient population and selection criteria

**Figure 1:**
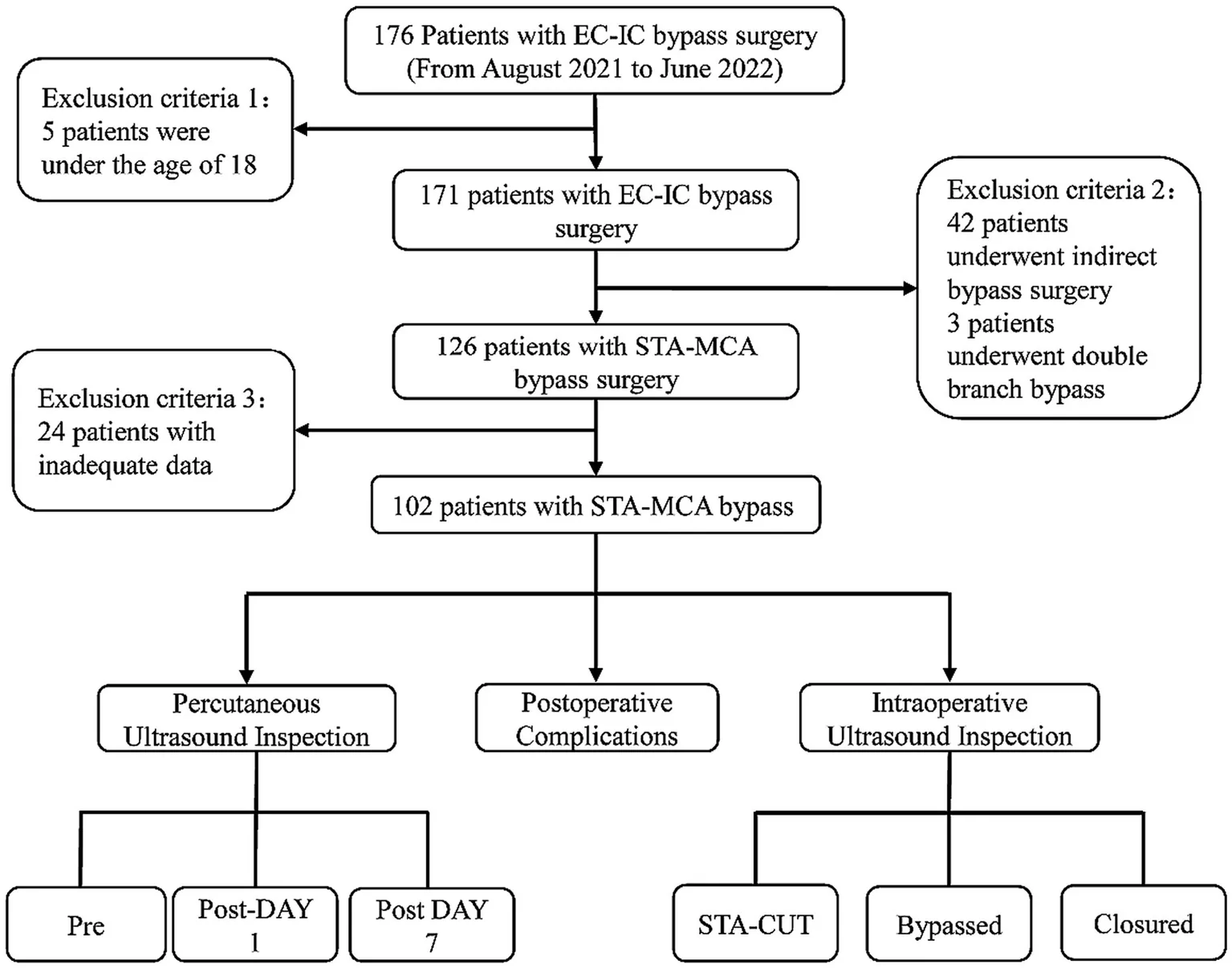
Flow diagram of the study population.

This retrospective study included all extracranial-intracranial (EC-IC) bypass procedures performed at our medical center for the treatment of cerebral ischemia secondary to cerebrovascular occlusive disease. The study spanned from August 2021 to June 2022, covering a one-and-a-half-year period with 6 months of postoperative follow-up. Cases where bypass surgery was performed for blood flow replacement during aneurysm or tumor surgery were excluded (Figure 1). The Institutional Review Board of Nanfang Hospital (NFEC-2022-442) approved the study, and informed consent was obtained from all participants.

### Surgical technique

All procedures were superficial temporal artery to middle cerebral artery (STA-MCA) bypasses. In brief, the bypass was performed using end-to-side anastomosis of the STA to the MCA, targeting the frontal and/or temporal M4 branches of the MCA. A 10-cm segment of the frontal and/or parietal branch of the STA was dissected, followed by craniotomy. After opening the dura, one or two MCA branches (for double-barrel bypass) were selected as recipient vessels and occluded with two temporary clips placed 1 cm apart. A longitudinal incision was made in the recipient vessel, irrigated with heparin solution, and the donor STA was trimmed obliquely to match the recipient artery. The anastomosis was completed with 8–12 interrupted sutures (3–5 per side). The patency of the anastomosis was verified using indocyanine green (ICG) video angiography, performed with a commercially available microscope (OPMI Pentero, Carl Zeiss Co, Oberkochen, Germany). During the surgery, non-invasive systolic blood pressure was controlled within the range of 110–130 mmHg, with the exception of the pressor test period. Meanwhile, end-tidal carbon dioxide (ETCO2) was maintained at 35–40 mmHg.

### Blood flow measurements

Intraoperative blood flow was measured using the MiraQ transit time ultrasonic flowmeter (TTFM) device (Medistim, Oslo, Norway). TTFM uses two ultrasonic transducers fixed to a flow probe, which transmit and receive ultrasonic signals across a fluid-filled vessel. The difference between upstream and downstream transit times of the ultrasound beam correlates with flow velocity. The transit time difference is directly proportional to blood flow volume. Mean graft flow (MGF = Qmean; ml/min) depends on graft quality, diameter, recipient vessel quality, and distal runoff. The pulsatility index (PI) estimates resistance in the graft and target vessel, calculated as the difference between peak systolic and peak diastolic flow divided by the mean flow (PI = [Qmax − Qmin]/Qmean) (31).

The flowmeter probe was placed on the graft vessels during surgery, ensuring 50% contact between the probe and the graft. Subsequent to the STA-MCA anastomosis and achievement of hemostasis (STA-Bypassed), and prior to galea closure, a 30-s measurement was performed to record the intraoperative graft flow and PI value (STA-Closured). If graft flow was insufficient, the anastomosis was revisited to rule out technical issues, and the final TTFM readings were used. To minimize blood loss, blood flow was measured for 10s following the release of the temporary clip on the transected distal end of the STA (STA-Cut).

Postoperative bypass function was monitored using quantitative ultrasonography in all patients. At our hospital, Toshiba Aplio 500 machines, duplex ultrasonography, and B-mode imaging were used to evaluate STA grafts. Patients were examined in the supine position, maintaining an incident angle between the STA and Doppler beam of 60 degrees or less. For single bypasses, flow was measured at the segment where the STA passes through the skull. For double-barrel bypasses, measurements were taken 3–5 mm proximal to the bifurcation of the frontal and parietal STA branches. Ultrasound examinations were performed preoperatively, on preoperative, postoperative day 1, day 7.

### Diagnosis of postoperative CHS

The diagnostic criteria for postoperative CHS were as follows: (1) Focal hyperperfusion in the territory of the postoperative graft was identified on arterial spin labeling (ASL) or CT perfusion imaging, without evidence of new cerebral infarction or intracranial hemorrhage; (2) The clinical manifestations corresponded closely to the anatomical site of hyperperfusion; (3) The aforementioned clinical symptoms were distinct from the preoperative presentation. MCA Recipient Network Grading Scale (MBC Scale).

In contrast to the Suzuki stage, which assesses the progression of moyamoya disease, the MCA recipient network grading scale (MBC scale) evaluates the intracranial blood flow compensation based on digital subtraction angiography (DSA) findings. The scale was scored as follows: (1) M1 lumen (M): 0, normal; 1, stenosis; 2, occlusion. (2) MCA bifurcation (B): 1, the MCA bifurcation and the communication between the blood vessels are normal; 0, MCA bifurcation occlusion. (3) cortical vascular integrity (C) 3, more than two-thirds of the MCA cortical arterial branches can be visualized on preoperative DSA; 2, one-third to approximately two-thirds can be visualized; 1, less than one-third can be visualized. Detailed interpretations of the MBC scale can be found in our previous study (16). A score of ≤4 indicates a poor intracranial vascular network, while a score of ≥5 suggests a good network.

### Statistical methods

Continuous variables are presented as mean ± standard deviation (SD). The student’s *t*-test was employed to assess differences in mean values between the well-recovery and complication groups. To explore the relationship between various indices [e.g., vessel diameter, blood flow, pulsatility index (PI), resistance index (RI)] and clinical factors such as Suzuki stage and MCA score, a logistic regression model using generalized estimating equations (GEE) was applied. Measurements were taken at five time points: baseline (Pre), postoperative day 1 (D1), and day 7 (D7). The differences between time points, including ΔD1-Pre and ΔD7-D1, were also analyzed. For repeated measures data, an autoregressive (AR) correlation matrix was utilized. Odds ratios (OR) were derived from the logistic regression coefficients. A *p*-value of <0.05 was considered statistically significant. All statistical analyses were conducted using GraphPad Prism version 9.3.1 (GraphPad Software, San Diego, CA, United States).

## Results

### Patients and operative demographics

A total of 102 patients were included in this cohort. The average age of the patients was 46.83 ± 11.34 (range, 19–66 years), and the sex ratio was 0.96:1 (male/female, =50/52). Thirty-three patients (32.35%) were undergoing combined revascularization surgery, while 69 cases (67.65%) were performed direct revascularization methods. Left hemisphere bypass surgery was performed in 49 cases, while right hemisphere bypass surgery was conducted in 53 cases. The characteristics of the 102 patients included in this study are shown in Table 1.

**Table 1:** The characteristics of 102 patients were included in regression analysis.

Parameters	Mean ± SD	*N*(%)	Median (IQR)	Range (min, max)
Age	46.83 ± 11.34		50.50 (38, 56)	47 (19,66)
Height	161.99 ± 6.77		162 (156, 168)	28 (148,176)
Weight	62.64 ± 9.75		63 (55, 70)	44 (41,85)
BMI	23.84 ± 3.44		23.51 (21.35, 26.13)	18.67 (17.36,36.03)
Sex				
Male		50 (49.02%)		
Female		52 (50.98%)		
Side				
Left		49 (48.04%)		
Right		53 (51.96%)		
Surgical approach				
Direct bypass		69 (67.65%)		
Combined bypass		33 (32.35%)		
MBC Scale				
≤4		50 (49.02%)		
≥5		52 (50.98%)		
Cerebral Hyperperfusion Syndrome (CHS)				
Yes		13 (12.75%)		
No		89 (87.25%)		

### Ultrasonography indices of STA over time

The STA flow and PI values measured by intraoperative ultrasound and non-invasive ultrasound at different time points are shown in Table 2.

**Table 2:** The results of ultrasonography indices for the bypass side.

Hemodynamic parameter	Intraoperative ultrasound			Non-invasive ultrasound		
	Cut	Bypassed	Closured	Pre	Day 1	Day 7
Flow	59.75 ± 37.49	36.07 ± 22.59	35.14 ± 22.93	7.06 ± 3.30	111.9 ± 62.06	104.5 ± 64.93
PI	0.89 ± 0.81	1.13 ± 0.74	1.28 ± 1.91	1.53 ± 0.39	0.84 ± 0.58	1.20 ± 1.91

### The graft arteries transition from low-flow, high-resistance to high-flow, low-resistance

**Figure 2:**
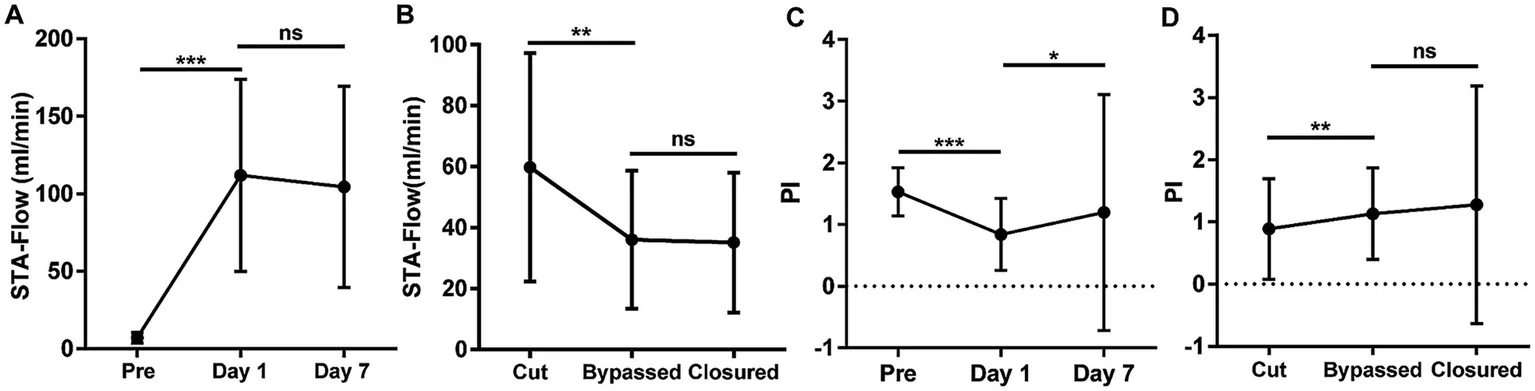
Trends of STA flow and PI in different periods. **(A)** Non-invasive quantitative ultrasound measurement of superficial temporal artery (STA) flow at preoperative, postoperative day 1 and postoperative day 7 time points; **(B)** Non-invasive quantitative ultrasound measurement of STA pulsatility index (PI) at preoperative, postoperative day 1 and postoperative day 7 time points; **(C)** Intraoperative ultrasound measurement of STA flow at cut, bypassed and galea-closed surgical stages; **(D)** Intraoperative ultrasound measurement of STA PI at cut, bypassed and galea-closed surgical stages. ns, non-sense; *p < 0.05, **p < 0.01, ***p < 0.001.

Figure 2 presents the trends of STA flow and PI across different time points. Preoperatively, the average STA flow was only 7.06 mL/min (Pre). In contrast, the averages rose significantly to 111.9 mL/min and 104.5 mL/min on postoperative day1 and day 7, respectively (Figure 2A). Meanwhile, the PI demonstrated a significant decrease postoperatively (Figure 2C). In the decrease group, fewer patients had MBC scores ≥5 compared to those with scores ≤4, while the opposite pattern was markedly observed in the increase group. Intraoperative ultrasonography revealed that transection of the STA resulted in a high-flow, low-resistance pattern due to the absence of distal resistance. However, following anastomosis and prior to skin closure, a significant decrease in flow was observed, accompanied by a rise in the PI (Figures 2B,D).

### The influence of the intraoperative blood pressure elevation on STA flow and PI

**Figure 3:**
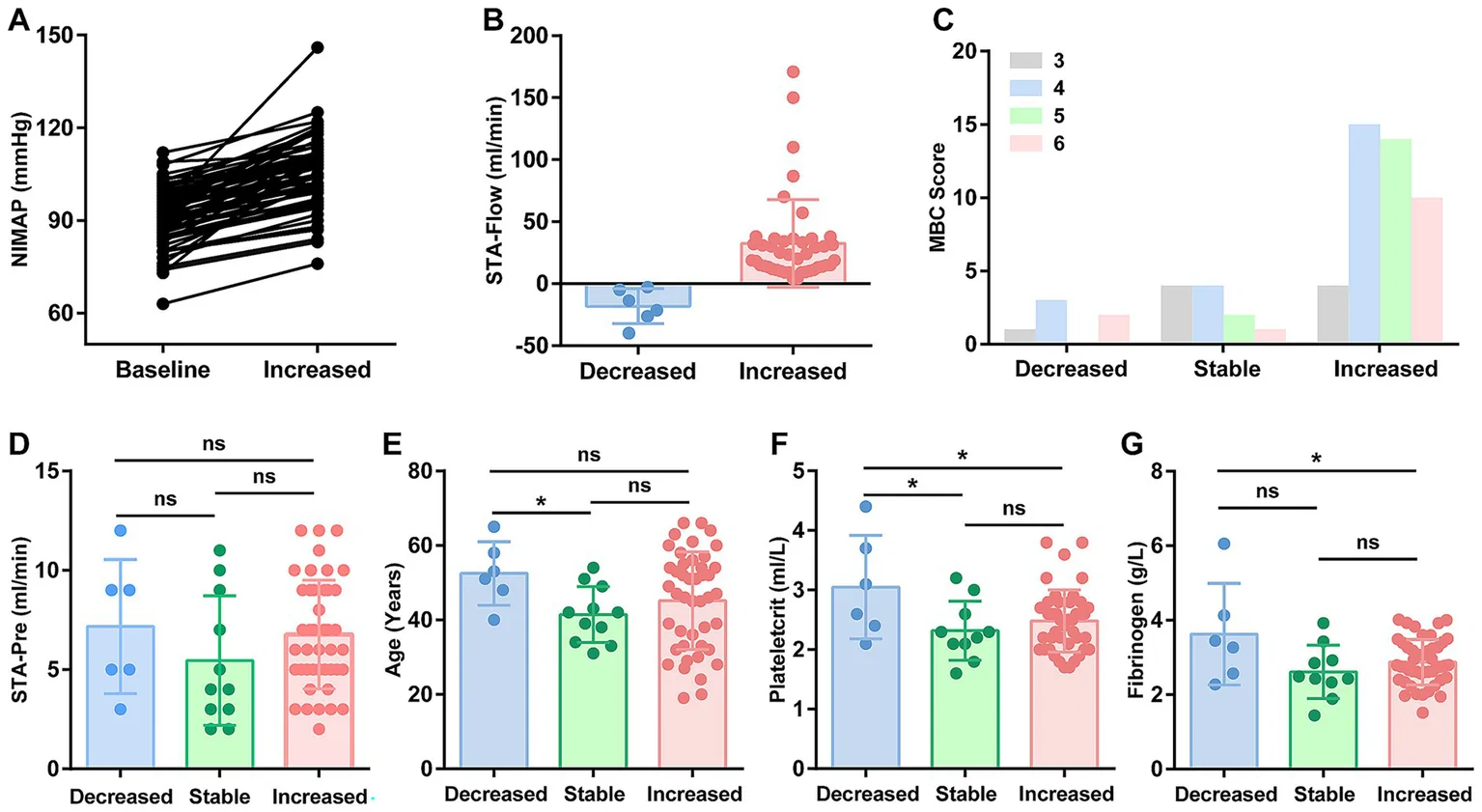
Effect of mean arterial pressure on STA hemodynamics. **(A)** Impact of vasopressors on blood pressure. **(B)** Effect of intraoperative blood pressure elevation test on STA-flow. **(C–G)** Factors influencing graft blood flow dynamics. *p < 0.05.

A total of 60 patients underwent the intraoperative pressor test. Intraoperatively, the patient’s mean arterial pressure (MAP) was elevated by a target of 15% (an actual increase of 16.09%, Figure 3A). The results showed that a decrease in blood flow was observed in 6 patients, an increase was seen in 43 patients, and no change occurred in 11 patients (Figure 3B).

### Factors influencing intraoperative changes in STA flow

We categorized the blood-pressure elevation test results into three groups based on flow characteristics: increased group, stable group, or decreased group. In the decreased group and stable group, fewer patients had MBC scores ≥5 compared to those with scores ≤4, while the opposite pattern was observed in the increase group (Figure 3C). Comparative analysis revealed that the decreased flow group exhibited significantly higher values for age, plateletcrit, and plasma fibrinogen compared to stable group or increased group (Figures 3E–G and Table 3). However, no statistically significant differences were observed among the groups in preoperative STA Flow (Figure 3D and Table 3).

**Table 3:** Factors influencing intraoperative changes in STA flow.

Parameters	Decreased group	Stable group	Increased group
MBC Scales (N)			
≤4	4	8	19
≥5	2	3	24
STA-Pre	15.17 ± 3.97	13.18 ± 2.68	15.98 ± 4.96
Age	52.50 ± 8.55	41.45 ± 7.50*	45.18 ± 13.10
Plateletcrit	3.05 ± 0.87	2.32 ± 0.50*	2.48 ± 0.52*
Fibrinogen	3.63 ± 1.36	2.61 ± 0.71	2.87 ± 0.61*

*Compared with the decrease group, *p* < 0.05.

### Influencing factors for cerebral hyperperfusion syndrome (CHS) after EC-IC bypass surgery

**Figure 4:**
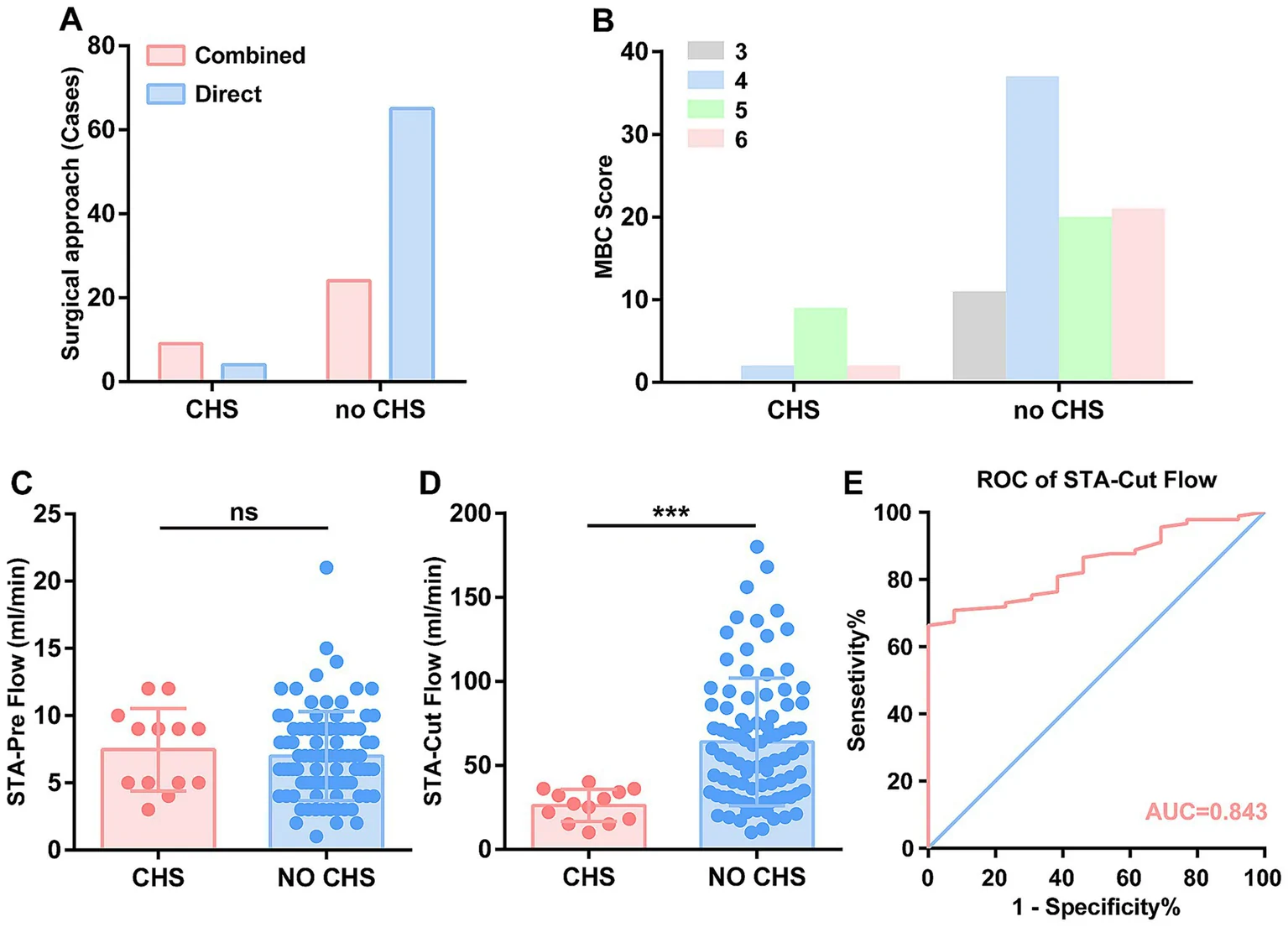
Risk factors for cerebral hyperperfusion syndrome (CHS). Logistic regression analysis of factors influencing CHS development. Factors associated with CHS included: **(A)** surgical approach, **(B)** MBC scale, **(C)** STA-pre flow, **(D)** STA-Cut flow. **(E)** ROC curve analysis to evaluate the predictive value of the STA-Cut flow for CHS. ***p < 0.001.

In our study cohort, the incidence of CHS following EC-IC bypass surgery, as diagnosed through standardized examination protocols, was 12.74% (13/102 cases). The proportion of combined bypass was significantly higher than that of direct bypass in the CHS group, while the reverse distribution was observed in the no CHS group (9/13 vs. 24/89, *p* = 0.004, Figure 4A). A significantly higher proportion of patients had an MBC score ≥5 compared to those with a score ≤4 in the CHS group, while the inverse distribution was observed in the no CHS group (11/13 vs. 41/89, *p* = 0.015, Figure 4B). No significant difference was observed in preoperative STA flow between the two groups (7.46 ± 3.07 vs. 6.99 ± 3.31, *p* = 0.629, Figure 4C). The intraoperative STA-Cut flow was significantly higher in the no CHS group than in the CHS group (26.15 ± 9.54 vs. 63.88 ± 38.01, *p* = 0.0006, Figure 4D). Receiver operating characteristic (ROC) curve analysis was performed to evaluate the predictive value of the STA-Cut Flow for CHS. The result revealed an area under the curve (AUC) of 0.843 (95%CI: 0.761–0.926, *p* < 0.001, Figure 4E), indicating that the STA-Cut value holds predictive significance for CHS. The cut-off value was 40.5 mL/min, with a sensitivity of 66.3% and a specificity of 100%.

## Discussion

This study demonstrates time-dependent hemodynamic alterations in the grafts of patients with moyamoya disease who underwent extracranial-intracranial bypass surgery. These alterations in STA flow may suggest different compensatory patterns of the vascular network (17). Accordingly, we believe that intraoperative ultrasonographic monitoring, with preoperative and postoperative Doppler ultrasonography, may predict the occurrence of adverse postoperative complications.

The alterations in the ultrasonographic parameters of STA shown that the flow of the graft would increase significantly after the operation. The features of the graft’s hemodynamics would convert from the original type (high-resistance and low output) to a compensatory type (low-resistance and high output) during the postoperative period, which is the root cause of hemodynamic changes (18). As time progressed, the graft adapted to the growth of collateral compensation, and hemodynamics began to stabilize. This is mainly related to the resistance changes in the vascular network distal to the graft. The STA originally coursed through the galea aponeurosis and subcutaneous fascia to supply the scalp and had a typical high-resistance terminal distal vessel. After separation and displacement from under the skin flap, the STA was sutured to the cerebral cortical artery. The distal vascular network of the STA immediately transformed into a low-resistance middle cerebral artery vascular network which suffered from long-term ischemia (18–20).

We evaluated factors affecting superficial temporal artery cut-off blood flow and graft blood flow. STA blood flow status, including cut flow and graft flow, is related to blood pressure and coagulation status (21). Blood flow decreases when blood pressure decreases or blood becomes viscous. In the management of extracranial intracranial bypass surgery patients, we should pay more attention to their blood pressure, blood lipids and coagulation function indicators. We are committed to finding a balance between maintaining normal coagulation function and avoiding graft anastomotic thrombosis.

In this study, we propose a novel grading scale for the assessment of long-term ischemic vascular networks, namely MCA recipient network grading scale (MBC scale). MBC scale reflects the vascular network of the recipient region by considering the M1 segment, bifurcation and cortical artery of the middle cerebral artery (MCA). A higher MBC scale score indicates a better cerebral vascular network in the recipient area. In the past, we often use Suzuki stage to judge the progress of moyamoya disease, but we believe that only referring to Suzuki stage is not comprehensive. Some patients with moyamoya disease have progressed to the end stage but still have a well-compensated vascular network. These patients can quickly relieve from sufferings of cerebral ischemia after surgery. Meanwhile, some MMD patients with Suzuki stage 2 or 3 do not have sufficient cortical arterial compensation, and these patients usually need more time to recover. The combined evaluation of MBC scale and Suzuki stage is helpful to understand the prognosis of patients.

The blood flow of organs in the body generally depends on the metabolic activity of organ tissues. Cerebral blood flow can be stable within a certain range of blood pressure changes. Cerebral blood flow should be increased or maintained in a short period of time after the increase of blood pressure, and the decrease of blood flow reflects the abnormal regulation of blood flow. According to Poiseuille’s law, blood flow is related to blood viscosity coefficient, vessel diameter, and blood pressure. On the basis of the elevated blood pressure test, we found that the patient’s increased age, decreased vascular elasticity, and blood status contributed to the abnormal blood flow regulation. Therefore, more attention should be paid to the management of blood pressure and blood status during the perioperative period (22–24).

Cerebral hyperperfusion syndrome (CHS) is a serious neurological complication characterized by postoperative deficits resulting from abnormally elevated cerebral blood flow (CBF) following revascularization surgery, particularly in adult patients with MMD (25, 26). According to a recent meta-analysis, the incidence of CHS in adults ranges from 11.7 to 29.4%, with a pooled estimate of 19.9% (27). CHS usually caused transient neurological impairment which usually completely relieved in 7–14 days after operation without permanent cerebral damage (7, 28). However, it should be noted that intracranial hemorrhage and subarachnoid hemorrhage secondary to CHS may lead to serious consequences (29–31). We explored the association of ultrasound parameters, MBC scale and Suzuki stage with CHS. After including all potential factors in binary logistic regression analysis, we found that low STA-Cut flow, high MBC scale score, and combined revascularization were associated with the development of CHS. We speculate that a high MBC score reflects chronic cerebral hypoperfusion, leading to compensatory dilation and impaired autoregulation of cerebral small vessels. Given the structural and functional homology between the STA and MCA cortical branches, similar changes occur in the STA, manifesting as low STA-Cut flow. Thus, even a modest increase in post-bypass flow may overwhelm the maximally dilated recipient vasculature and trigger CHS. When encountered in patients with low STA-Cut flow and high MBC scale score, we should intervene as early as possible. As for surgery approach, previous studies did not find a difference in the incidence of CHS between direct and combined bypass. The emergence of positive results in this study may be due to cortical vasospasm after temporal muscle application combined with revascularization, and the donor artery blood flow could not be relieved.

This study has the limitations inherent to any single institution study. While patients were selected for bypass based on institutional protocols and guidelines and multidisciplinary consensus, there exists the possibility of selection bias. Intra-observer variation is a potential problem in ultrasound examinations. Despite these considerations, the ultrasonography during bypass procedures in the study were performed by 2 experienced cerebrovascular surgeons, and the method of recording intraoperative blood flow measurements remained similar over the study period.

## Conclusion

In extracranial intracranial revascularization, intraoperative ultrasonographic findings of the STA correlated well with the extent of prognosis. After extracranial intracranial bypass surgery in patients, the hemodynamics of the STA changes as the graft adapts and compensates. Early intervention should be considered for patients with low MBC scale score assessed by cerebral angiography preoperatively and cutoff flow decrease on intraoperative ultrasound.

## Data Availability

The raw data supporting the conclusions of this article will be made available by the authors, without undue reservation.
